# Supplementary material for: The role of the Big Two in socially responsible behavior during the COVID-19 pandemic: Agency and communion in adolescents’ personal norm and behavioral adherence to instituted measures
Source: PLoS One. 2022 Jun 9;17(6):e0269018. doi: 10.1371/journal.pone.0269018 (PMC9182629; doi:10.1371/journal.pone.0269018)
Supplement: S1 Table — (DOCX) [file pone.0269018.s001.docx]

| **Table S1. Communalities and results of unrotated and rotated maximum likelihood analysis**  **(factor loadings)** | | | | | |
| --- | --- | --- | --- | --- | --- |
|  |  | Agency | | Communion | |
|  | *Communality* | *Unrotated* | *Rotated* | *Unrotated* | *Rotated* |
| C1 (emotional) | 0.53 | -0.46 | -0.11 | 0.56 | 0.76 |
| C2 (romantic) | 0.20 | -0.20 | 0.04 | 0.40 | 0.43 |
| C3 (industrious) | 0.14 | 0.04 | 0.24 | 0.37 | 0.21 |
| C4 (sympathetic) | 0.31 | -0.27 | 0.03 | 0.49 | 0.55 |
| C5 (empathetic) | 0.55 | -0.45 | -0.08 | 0.59 | 0.77 |
| A1 (companionable) | 0.23 | 0.02 | 0.29 | 0.48 | 0.31 |
| A2 (humorous) | 0.16 | 0.11 | 0.31 | 0.38 | 0.17 |
| A3 (courageous) | 0.53 | 0.54 | 0.75 | 0.49 | -0.10 |
| A4 (sporty) | 0.31 | 0.37 | 0.56 | 0.42 | -0.02 |
| A5 (strong) | 0.54 | 0.54 | 0.76 | 0.50 | -0.10 |
| C1-C5 – communion items; A1-A5 – agency items | | | | | |
