# Supplementary material for: The role of the Big Two in socially responsible behavior during the COVID-19 pandemic: Agency and communion in adolescents’ personal norm and behavioral adherence to instituted measures
Source: PLoS One. 2022 Jun 9;17(6):e0269018. doi: 10.1371/journal.pone.0269018 (PMC9182629; doi:10.1371/journal.pone.0269018)
Supplement: S2 Table — (DOCX) [file pone.0269018.s002.docx]

| **Table S2. Communalities and results of unrotated and Oblimin-rotated principal component analysis (factor loadings)** | | | | | |
| --- | --- | --- | --- | --- | --- |
|  |  | Agency | | Communion | |
|  | *Communality* | *Unrotated* | *Rotated* | *Unrotated* | *Rotated* |
| C1 (emotional) | 0.64 | -0.57 | -0.05 | 0.56 | 0.80 |
| C4 (sympathetic) | 0.55 | -0.44 | 0.06 | 0.60 | 0.73 |
| C5 (empathetic) | 0.72 | -0.57 | -0.01 | 0.63 | 0.85 |
| A3 (courageous) | 0.67 | 0.56 | 0.82 | 0.60 | -0.02 |
| A4 (sporty) | 0.53 | 0.47 | 0.73 | 0.56 | 0.03 |
| A5 (strong) | 0.69 | 0.57 | 0.83 | 0.60 | -0.02 |

C1-C5 – communion items; A1-A5 – agency items
