# Supplementary material for: The role of the Big Two in socially responsible behavior during the COVID-19 pandemic: Agency and communion in adolescents’ personal norm and behavioral adherence to instituted measures
Source: PLoS One. 2022 Jun 9;17(6):e0269018. doi: 10.1371/journal.pone.0269018 (PMC9182629; doi:10.1371/journal.pone.0269018)
Supplement: Attachment — Submitted filename: Response to Reviewers.docx [file pone.0269018.s003.docx]

**MS number**: PONE-D-22-00800

**Title**: The role of the Big Two in socially responsible behavior during the COVID-19 pandemic: Agency and communion in adolescents’ personal norm and behavioral adherence to instituted measures

Dear Reviewer,

Thank you very much for your time and valuable review. Your and Editor’s feedback improved our manuscript. Please find below our responses on your comments and recommended actions.

Sincerely,

The authors

**Reviewer 1**

**1. I was a bit surprised to read in the Method section how “personal norm” was operationalized. I expected somewhat different from reading the Introduction. Perhaps the authors could clarify early on in the manuscript how precisely they use the term “personal norm” in their research.**

Thank you very much for this remark. We indicate throughout the manuscript now how exactly we use the term “personal norm”:

In the abstract:

“In this article, we focus on adolescent boys’ and girls’ agency and communion orientations to explain the level of importance they attribute to the instituted measures to contain the spread of the COVID-19 virus *(personal norm)*, as well as their behavioral adherence to those measures.”

In the introduction (page 3):

“In this article, we focus on adolescent boys’ and girls’ agency and communion orientations to explain the level of importance they attribute to the instituted measures to contain the spread of COVID-*19 – their personal norm*, as well as their behavioral adherence to those measures.”

In the theoretical part (page 6):

“Thus, the COVID-19 situation could allow the expression of both agency and communion, as both dimensions might play a positive role in personal norm *– perceived importance of* and behavioral adherence to the instituted measures.”

In the discussion (page 16):

“The main goal of this study was to investigate the role of agency and communion in boys’ and girls’ personal norm regarding *the importance of* the instituted measures to contain the spread of COVID-19 virus, as well as their behavioral adherence to those measures. Although it was assumed that in the pandemic context both agency and communion might be positively related to personal norms regarding *the importance of the* COVID measures and behavioral adherence to them, our results showed only communion as a positive predictor and agency as a negative predictor of those aspects of socially responsible behavior among adolescents.”

**2. Good decision to drop some items. They not only evidenced poor psychometric properties, but also poor face validity (IMO).**

We also agree that the face validity of excluded items was low.

**3. Are the effects of agency and communion unique effects? That is, is the shared variance between agency and communion pulled out statistically? Figure 1 suggests that it is not. Research on the Big Two and norm conformity, however, typically focuses on those unique effects (e.g., Gebauer, Paulhus, & Neberich, 2013, SPPS).**

Thank you for this question. In our analyses, both agency and communion were included in the models together in order to control for the influence of one variable (e.g., agency) while testing the predictive role of another variable (e.g., communion). This way, our analytic procedure is in line with Gebauer et al. (2013) in which they emphasize the step of controlling for one over the effect of another Big Two dimension. Moreover, the correlation between agency and communion in our sample is small to moderate (r = .289, p <.001), similar to the study of Gebauer et al. (2014; r = .34). To examine potential problems regarding multicollinearity, we computed the VIF for agency and communion, yielding the following results:

VIF (agency) = 2.08

VIF (communion) = 1.49

Generally, VIFs higher than 5 are considered indicative of collinearity, suggesting potential difficulties in separating out the independent contribution of the variables concerned (James et al., 2013). Other authors suggest a more conservative approach, considering VIFs greater than 2.5 indicative of collinearity (Johnston et al., 2018). Even if the stricter criterion is applied, our results suggest absence of any difficulties in separating out the independent contribution of agency and communion, with current results indicating their unique effects.

If there are any additional steps and procedures we can conduct to answer your question and assure adequate analytic procedure, we would be more than happy and eager to implement them.

P.S.: In line with the result of a significant correlation between our predictors, factor correlation has been specified in our Figures.

**4. The negative relation between agency and norm adherence is described as counter to expectations. However, from the perspective of extant research on the Big Two and conformity to sociocultural norms this negative relation is the expected one (if anything, the very small size of that relation is surprising) (for a review, see Gebauer, Lüdtke, Sedikides, & Neberich, 2014, JOPY)**

We are aware of research conducted by Gebauer and colleagues. In fact, our study is based on Bakan’s expressiveness perspective that they apply and expand in their research. According to both Bakan’s perspective and Gebauer et al.’s research, as you suggested, it is the expression of communion and not agency that is allowed in the context of prosociality. In our theoretical reasoning we used that same starting point. However, taking into account the fact that studies on adolescents’ prosocial behavior (e.g., Eberly-Lewis & Coetzee, 2015), as well as context-specificity of pandemic, both suggest that socially responsible behavior in the COVID-19 pandemic can be driven by both intentions to protect others form the virus (communion) and intention to protect oneself from the virus (agency), we hypothesized that the context-specificity of our data collection allows, in fact, expression of both agency and communion. Moreover, studies within the same COVID-specific context showed an increase in self-oriented motives (agency) as a compensatory mechanism (Zhao et al., 2021), and indicated that participants mostly followed guidelines that protected themselves rather than the general public (Leder et al., 2021). Hence, positive relations of both agency and communion were expected in our study. This is why the negative relation between agency and norm adherence was described as counter to expectations.

We did, however, reflect more in detail on Gebauer et al.’s (2014) findings and connect it with ours in the discussion now (page 17):

“Similarly, this finding is, to some extent, in line with studies on prosocial behavior among adolescents [23], *as well as among adults [22]*, that showed communion as a positive, but agency as a negative factor. *Moreover, Gebauer et al. [22] found communion to be a stronger predictor of prosocial behavior in countries where interest in prosociality is common, and weaker in countries where such behavior is uncommon (communal social assimilation). In contrast, agency negatively predicted interest in prosocial behavior comparatively weakly in countries where interest in prosociality was common, and comparatively strongly in sociocultural contexts where such interest was uncommon (agentic social contrast). Applying that to our results, and insofar prosocial behavior can be equated to socially responsible behavior during the pandemic, this could indicate a stronger interest in prosociality in Austria where data was collected*.”

Your comment also made us realize that we previously cited wrong Gebauer et al.’s study in our manuscript. That is corrected now in the reference list (reference No. 22, page 24).

**References mentioned in the rebuttal letter “Response to Reviewers”:**

Eberly-Lewis, M. B., & Coetzee, T. M. (2015). Dimensionality in adolescent prosocial tendencies: Individual differences in serving others versus serving the self. Personality and Individual Differences, 82, 1–6. [https://doi.org/10.1016/j.paid.2015.02.032](https://psycnet.apa.org/doi/10.1016/j.paid.2015.02.032)

Gebauer, J. E., Paulhus, D. L., & Neberich, W. (2013). Big Two Personality and Religiosity Across Cultures: Communals as Religious Conformists and Agentics as Religious Contrarians. *Social Psychological and Personality Science, 4*(1), 21–30. <https://doi.org/10.1177/1948550612442553>

Gebauer, J. E., Sedikides, C., Lüdtke, O., & Neberich, W. (2014). Agency-communion and interest in prosocial behavior: social motives for assimilation and contrast explain sociocultural inconsistencies. *Journal of personality*, *82*(5), 452–466. <https://doi.org/10.1111/jopy.12076>

James, G., Witten, D., Hastie, T., & Tibshirani, R. (2013). *An Introduction to Statistical Learning* (Vol. 103). Springer. <https://doi.org/10.1007/978-1-4614-7138-7>

Johnston, R., Jones, K., & Manley, D. (2018). Confounding and collinearity in regression analysis: a cautionary tale and an alternative procedure, illustrated by studies of British voting behaviour. *Quality & Quantity, 52*(4), 1957–1976. <https://doi.org/10.1007/s11135-017-0584-6>

Leder, J., Pastukhov, A., & Schütz, A. (2020). Even prosocially oriented individuals save themselves first: Social Value Orientation, subjective effectiveness and the usage of protective measures during the COVID-19 pandemic in Germany. <https://doi.org/10.31234/osf.io/nugcr>

Zhao, L., Ding, X., & Yu, F. (2020). Public moral motivation during the COVID-19 pandemic: Analysis of posts on Chinese social media. *Social Behavior and Personality: An international journal*, *48*(11), e9829.
